# An alternative methodology for the determination of the radiochemical purity of ^11^C-methionine

**DOI:** 10.1186/s41181-018-0053-0

**Published:** 2018-12-27

**Authors:** Javier Giglio, Gabriela Rosas, Martín Basso, Andrea Boné, Eduardo Savio, Henry Engler

**Affiliations:** Uruguayan Centre of Molecular Imaging (CUDIM), Ricaldoni 2010, 11600 Montevideo, Uruguay

**Keywords:** [^11^C]methionine, Quality control, Radiochemical purity, Enantiomeric purity, Radiopharmaceutical, PET

## Abstract

**Background:**

[^11^C]Methionine ([^11^C]MET) acts as an oncological radiopharmaceutical for measuring increased protein synthesis in brain tumours while having low uptake in normal brain an. In several Latin American countries, the official pharmacopoeias are the United States of America Pharmacopoeia (USP) and the European Pharmacopoeia (EP). For determination of the radiochemical purity, we used the monograph of L-methionine injection ([^11^C] methyl-methionine) of the EP (01/2002: 1617) because it is not available in the USP.

We present herein an alternative methodology for the determination of the radiochemical purity, which allows the assessment of the [^11^C]CH_3_I and of the degradation radioactive products.

**Results:**

The proposed method enabled a greater number of impurities to be determined than could be identified using the EP method. Under the conditions proposed by EP, it is not possible to determine the presence of [^11^C]CH_3_I or other lipophilic radiochemical impurities in the final product.

For the determination of enantiomeric purity according to EP a chiral thin-layer chromatography (TLC) allows the determination of D and L methionine between the established limits. This is a slow method and presents an inadequate detection due to the decay of the sample. An alternative HPLC method was optimized.

**Conclusion:**

The proposed radiochemical purity method allowed the identification of a greater number of radiochemical impurities compared to the method proposed by the EP monograph in 16 min.

The proposed enantiomeric purity method enables to perform the determination in a reasonable amount of time.

A discussion concerning the limit of 95% radiochemical purity proposed by the EP is necessary. If more radiochemical impurities can be detected, it is more reasonable to propose a limit of 90% for this parameter to perform clinical examinations.

## Background

L-Methionine is an essential amino acid involved in multiple metabolic pathways. Many tumor cells have increased expression of amino acid transporters to cover the synthesis of proteins. Methionine participates in the synthesis of different proteins as donor of methyl groups and is a precursor of cysteine and derivatives. (Ishiwata et al., 1988) For these reasons, L-[methyl-^11^C]-methionine ([^11^C]MET) acts as an oncological radiopharmaceutical of increased protein synthesis in brain tumours having low uptake in a normal brain. (Jager et al., 2001; Cook et al., 1999) [^11^C]MET can be used in patients with gliomas to delineate uptake areas for surgery, radiotherapy, to guide biopsies, monitor and predict the response to treatments (Jacobs et al., 2005; Nuutinen et al., 2000). It has been used for other applications, such as hyperparathyroidism. (Rubello et al., 2006)

**Fig. 1:**
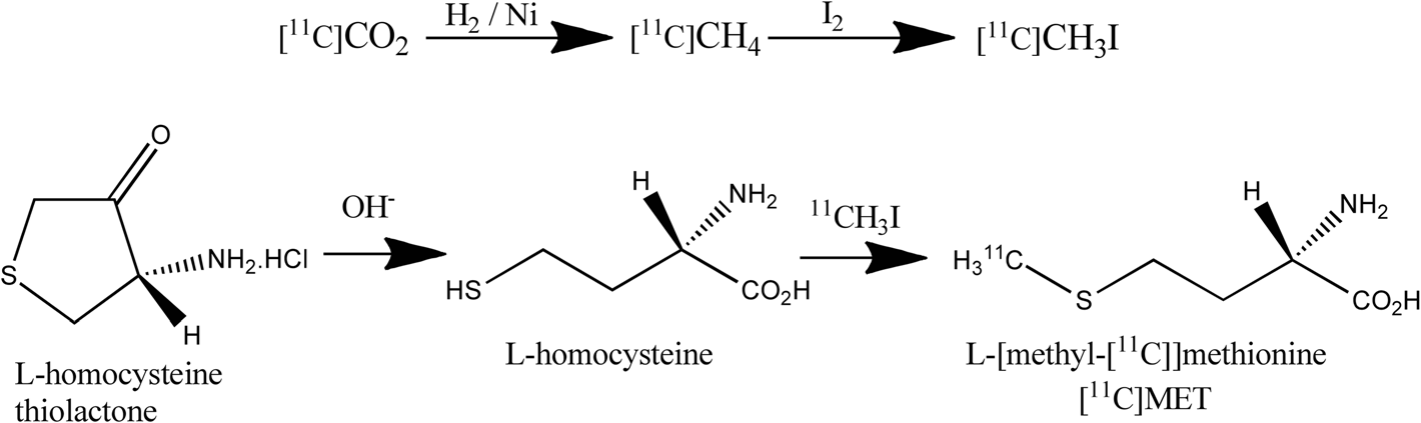
Scheme of the synthesis of [^11^C]MET

One of the first published [^11^C]-L-Methionine synthesis studies was conducted by B. Langström and H. Lundqvist (Långström & Lundqvist, 1976), who started with L-S-Benzyl homocysteine and made it react with [^11^C]CH_3_I in sodium and liquid ammonium. Currently, one of the most common synthesis routes for routine use in clinical environments is derived from the work of Pascali et al. (Pascali et al., 1999), in which the radioactive precursor [^11^C]CH_3_I is placed in a basic medium with homocysteine thiolactone in a solid support, according to Fig. 1.

This methodology is robust and allows producing the radiopharmaceutical in a reasonable amount of time. The method is being applied worldwide but there is great variation in terms of quality control between different centres. (Lodi et al., 2012)

The most important parameters to control are the radiochemical and enantiomeric purity. Although the synthesis uses the precursor with the correct chirality, it is possible for the inversion of the chirality to take place. Enantiomeric purity is related to the concentration of sodium hydroxide and the proportion of the solvents used. (Pascali et al., 1999; Gomzina & Kuznetsova, 2011) To ensure radiochemical purity, it is important that the system allows the detection of all the potentially present radiolabelled molecules. (Bogni et al., 2003; Woods et al., 2017)

In several Latin American countries, the official pharmacopoeias are the United States of America Pharmacopoeia (USP) (The United States Pharmacopeia (USP-40) and the National Formulary (NF-25), 2017) and the European Pharmacopoeia (EP). (Council of Europe, 2014) For determination of the radiochemical purity, we used the monograph of L-methionine injection ([^11^C] methyl) of the EP (01/2002: 1617) because it is not available in the USP.

We present herein an alternative methodology for the determination of the radiochemical purity, which allows the assessment of the [^11^C]CH_3_I and of the degradation radioactive products.

## Results and discussion

The [11C]MET used in this study was produced at the Uruguayan Molecular Imaging Center (CUDIM), through a reaction on a solid phase extraction cartridge.

### Radiochemical purity

According to the literature, radiochemical purity was determined using systems different from those outlined by the EP. An increase in the flow and in the run time were the main changes observed. Table 1 summarises some of the conditions used in different published studies.

**Table 1:** Quality control (QC) conditions reported in the literature and in the EP for the synthesis of [^11^C]MET with ultraviolet (UV) detection

Author/ year	Column	Eluent	Flow (mL/min)	Run time (min)	Homocys-teine (tr in min)	Homocys-teine thiolactone (tr in min)	Methi-onine (tr in min)
EP (Council of Europe, 2014)	C18 (250 × 4.6 mm, 5 μm)	KH2PO4 1.4 g/L	1.0	10	2.1	7.0	2.6
A.Bogni et al., 2003 (Bogni et al., 2003)	YMC-Pack pro C18 (4.6 × 250 mm 5 μm)	NaH2PO4 0.05 M	1.2	nr	nr	nr	nr
Mitterhauser et al., 2005 (Mitterhauser et al., 2005)	Phenosphere 10 μm ODS(1)	KH2PO4 1.4 g/L	1–4 min:1.0; 4–15 min: 3.0	nr	4.5	nr	5.2
G. Quinoces et al., 2006 (Quincoces et al., 2006)	Supelcosil C18 (250 × 4.6 mm, 10 μm)	KH2PO4 0.01 M	1.0	nr	nr	nr	3.0
F. Lodi et al., 2007 (Lodi et al., 2007)	Nucleosil 100–3 C18 (3 × 250 mm) M-N	NaH2PO4 0.05 M:EtOH (98:2)	0.3	nr	4.5	4.3	5.5
V. Gómez et al., 2008 (Gómez et al., 2008)	Zorbax eclipse SB-Aq C18 (4.6 × 250 mm, 5 μm)	KH2PO4 1.4 g/L	1.0	13	nr	nr	4.12
F. Lodi, et al., 2008 (Lodi et al., 2008)	Nucleosil 100–3 C18 (3 × 250 mm) M-N	NaH2PO4 0.05 M:EtOH (98:2)	0.3	nr	4.5	4.3	5.5
N. Gozmina, et al., 2011 (Gomzina & Kuznetsova, 2011)	Zorbax SCX (250 × 4.6 mm)	NaH2PO4 0.01 M pH = 3	1.0	nr	4.12	20.1	5.93
C. Pascali et al., 2011 (Pascali et al., 2011)	μBondapack C18 (300 × 3.9 mm, 10 μm)	NaH2PO4 0.003 M pH = 3.1	1.0	nr	3.34	3.70	3.80
	YMC-Pack Pro C18 (250 × 4.6 mm, 5 μm)	KH2PO4 0.05 M	1.0	nr	4.05	4.12	5.5
	YMC-Pack Pro C18 (250 × 4.6 mm, 5 μm)	KH2PO4 0.01 M	1.0	nr	3.94	3.68	5.55
	Spherisorb ODS1 (250 × 4.6 mm, 5 μm)	KH2PO4 0.01 M	1.0	nr	3.39	10.78	4.31

*nr* not reported

The system that was initially tested in our centre was the same as that outlined by the EP. The retention times of the different species depended on the C18 column, according to Pascali et al. (Pascali et al., 2011). The characteristics of the two columns used are shown in Table 2.

**Table 2:** Characteristics of the tested stationary phases

Column	Model	Size (mm)	Surface area (m^2^/g)	Pore size (nm)	Carbon loading (%)
EP characteristics	5 μm	250 × 4.6	220	8	6.2
Phenomenex (PH)	Phenosphere ODS(1) - 5 μm	250 × 4.6	220	8	7
Mancherey & Nagel (MN)	EC250/4.6 Nucleodur 100–5 C18ec - 5 μm	250 × 4.6	340	11	17.5

**Fig. 2:**
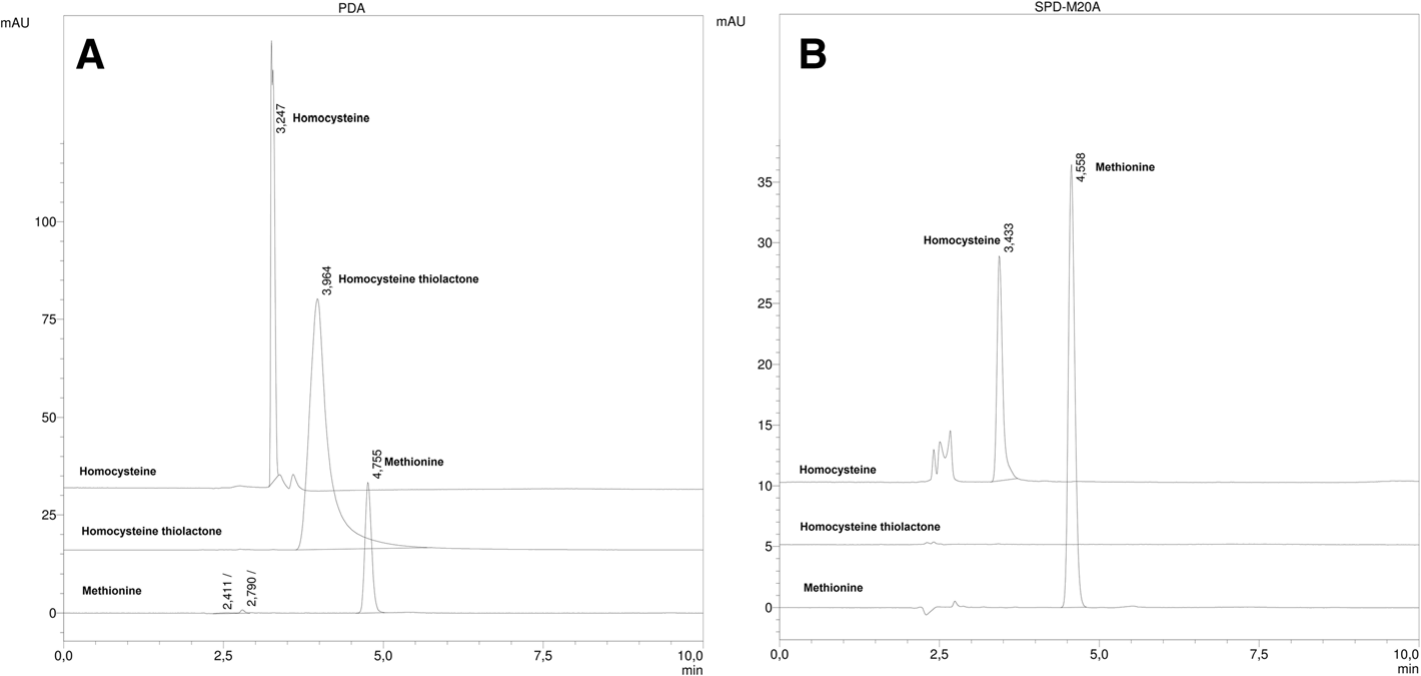
Chromatograms with the EP conditions for the reference solutions at UV 225 nm: **a**) Mancherey & Nagel (MN) column **b**) Phenomenex (PH) column

The chromatograms of the standard compounds L-homocysteine thiolactone (Impuriy a), L-Homocysteine (impurity b) and methionine at 225 nm in both columns used in the EP method are shown in the Fig. 2.

In the monograph for the L-Methionine in the EP, methionine is reported with a retention time of about 2.6 min, eluting after the peak of L-homocysteine and before the peak corresponding to the homocysteine thiolactone. Under this condition, different chromatographic profiles were obtained in the columns tested. In addition, the elution order of the peaks also changed. The resolution of the three species was achieved with the MN column, but the homocysteine thiolactone did not elute in the order described by the EP (eluting in the middle of homocysteine ​​and methionine). In the PH column, the homocysteine thiolactone peak was not observed during the run time (10 min), being retained inside the column.

**Fig. 3:**
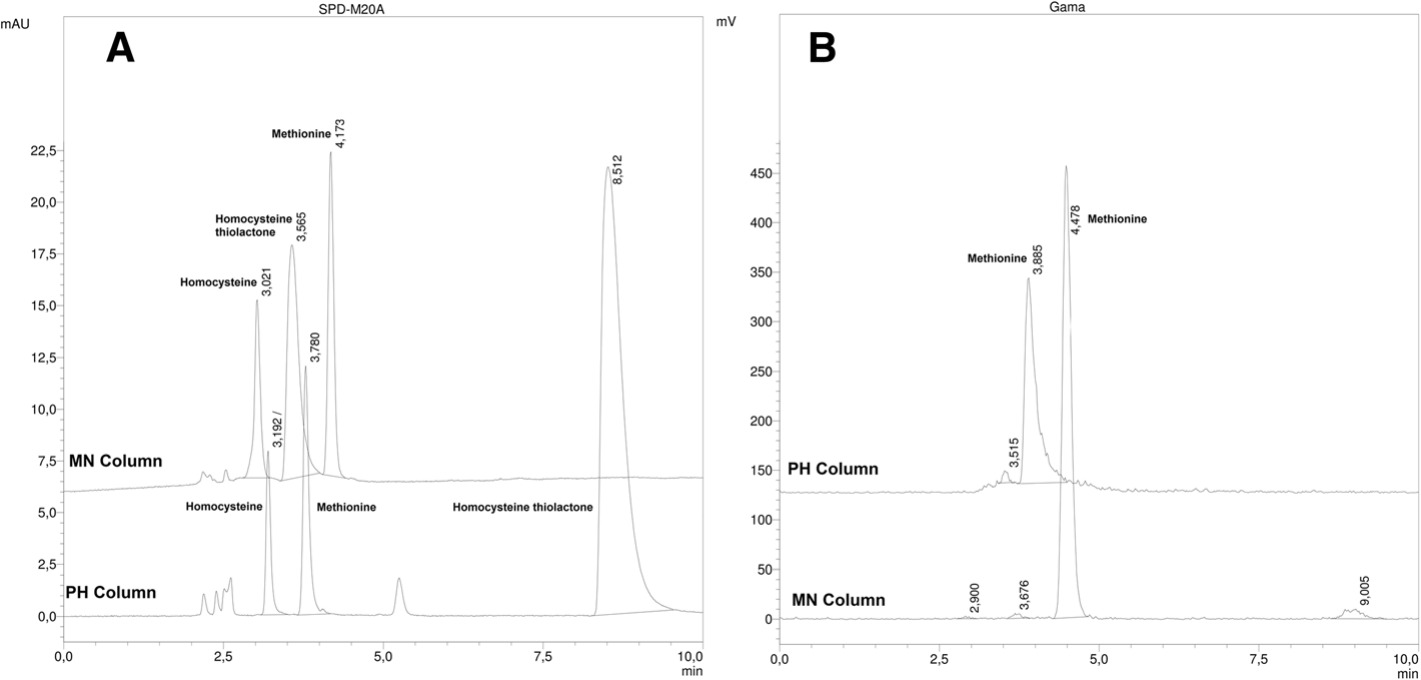
Chromatograms with a gradient of 1 to 3% CH_3_CN in 0 to 10 min: **a**) MN and PH column for reference solutions at UV 225 nm. **b**) MN and PH column for the sample (gamma detector)

In order to detect all the peaks of the reference solutions in the PH column, the lipophilicity of the mobile phase was increased. For this purpose, a gradient with acetonitrile (0 to 10 min gradient of 1 to 3% acetonitrile) was added. The chromatograms of the reference solutions in both columns under the new conditions in the same run time are shown in Fig. 3 a.

The modification previously described enabled the elution of the three peaks of the standard in both columns. However, the PH column showed a better resolution and the peaks eluted in the order established by the EP with the UV detector (see Fig. 3 a).

Different results were obtained when the same sample corresponding to the [11C]MET batch was analysed with gamma detection (see Fig. 3 b). For the MN column, three radiochemical impurities were observed, determining a radiochemical purity of 94,6%. For the same batch, using the PH column, a single radiochemical impurity was observed, with a radiochemical purity the 97,5%. These results were tested for different batches, determining always a higher radiochemical purity for the PH column compared to the MN column. The system with the MN column was selected for the determination of radiochemical impurities during the rest of the study using the [11C]MET samples. This is because the system using the MN column allowed the separation of a greater number of radiochemical impurities than the system with the PH column.

**Fig. 4:**
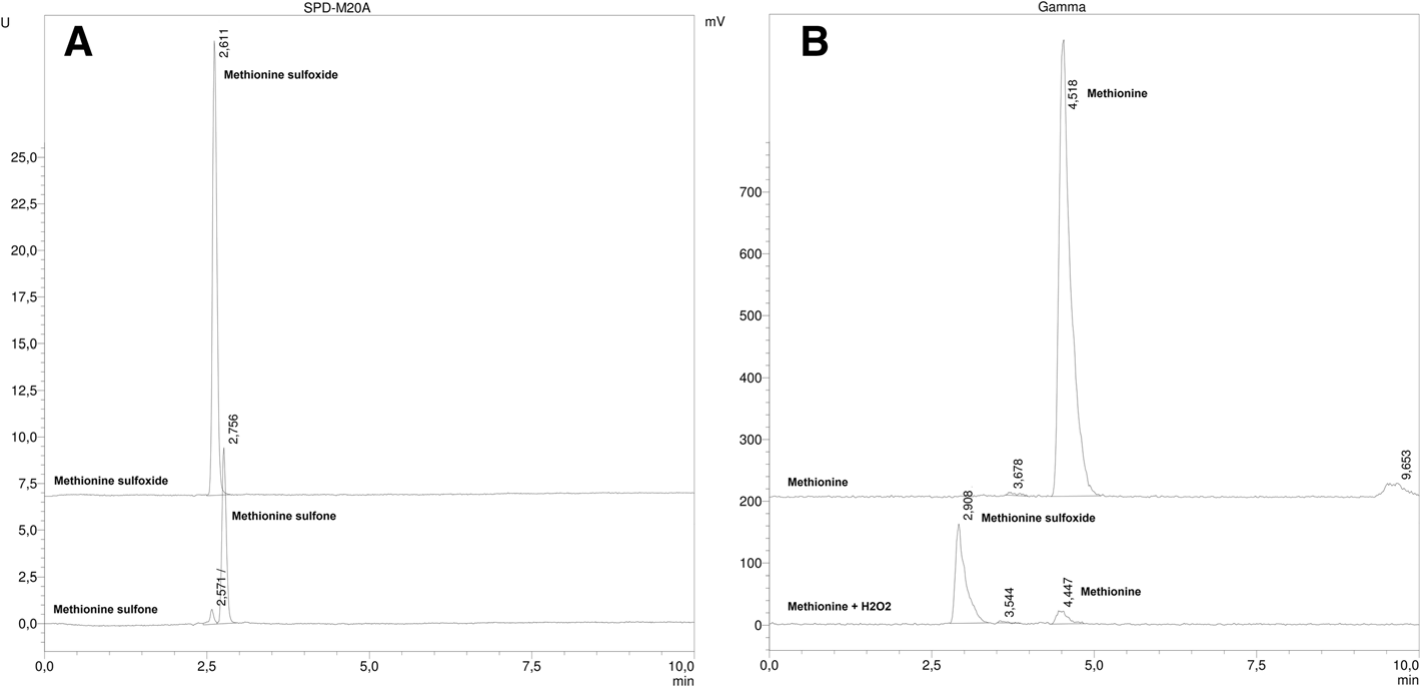
Chromatogram: **a**) reference solutions of methionine sulfoxide and methionine sulfone in the MN column (UV 225 nm). **b**) [^11^C]MET before and after treatment with H_2_O_2_, using gamma detector

The next step was to identify radioactive impurities, as the sulfoxide had already been checked, as reported by Bogni et al. (Bogni et al., 2003) and in the EP. Under our conditions, we detected two peaks (gamma detection) with shorter retention times than methionine, that we initially supposed to be the methionine sulfone (impurity d) and methionine sulfoxide (impurity c). To identify these peaks, the methionine sulfoxide and methionine sulfone reference solution were injected into the HPLC under the same conditions (i.e., an MN column with a gradient of 1 to 3% CH_3_CN). The chromatograms are shown in Fig. 4 a.

The oxidised species had a retention time of 2.61 and 2.76 min, not being possible to achieve the complete resolution of them. We hypothesized that the first peak may correspond to the oxidation compound of methionine (because to the time difference between the UV and gamma detector is 0.3 min) and the second one to an unknown impurity. In order to confirm this, a forced oxidation test of a methionine sample with hydrogen peroxide (H_2_O_2_) was carried out. After performing this assay, an increase in the peak with a retention time of 2.8 to 2.9 min was observed, but no changes in the peak at 3.5–3.6 min were observed. These experiments provide evidence that the peak corresponding to 2.9 min was the methionine sulfoxide and that the peak with a retention time of 3.5–3.6 min is an unknown impurity. The HPLC forced degradation is shown in Fig. 4 b.

The peak at 9 min in the HPLC of [^11^C]MET with gamma detector corresponded, according to Bogni et al., to unreacted [^11^C]CH_3_I (Bogni et al., 2003). A reference solution of CH_3_I was injected into the HPLC, but no peak was observed as the CH_3_I was retained by the column. The determination of [^11^C]CH_3_I must be done during the routine quality control because it is the alkylating agent in the reaction of the radiopharmaceutical. The EP request to check [^11^C]CH_3_OH but not [^11^C]CH_3_I. However the detection of [^11^C]CH_3_OH depends on the synthesis procedure. In case of using [^11^C]CH_3_OT_f_ it hydrolyses to methanol, while using [^11^C]CH_3_I this does not take place. We recommend that the EP should include both possibilities. We decided to modify the system to enable the determination of all the species present in the sample. A gradient up to 80% acetonitrile was used after the [^11^C]MET peak to elute the CH_3_I and avoid influences on the resolution of the observed peaks. The final conditions proposed for [^11^C]MET are shown in Table 3. The CH_3_I was not mentioned as a possible impurity by pharmacopoeia, despite being the radiosynthetic precursor most commonly used in the synthesis of methionine. The CH_3_I is a very lipophilic compound, needing to increase the lipophilicity of the mobile phase for its elution. In a C18 column, its elution was not possible under the conditions reported by the EP or by the authors mentioned in Table 1.

**Table 3:** The final HPLC conditions proposed

Column	Macherey-Nagel - EC250/4.6 NUCLEODUR 100–5 C18ec (MN)	
Flow	1.0 mL/min.	
Run time	16 min.	
Solvent A	KH2PO4 1,4 g/L	
Solvent B	CH3CN	
Gradient	% A	% B
0 to 6 min	99 to 98	1 to 2
6 to 8 min	98 to 20	2 to 80
8 to 16 min	20	80

**Fig. 5:**
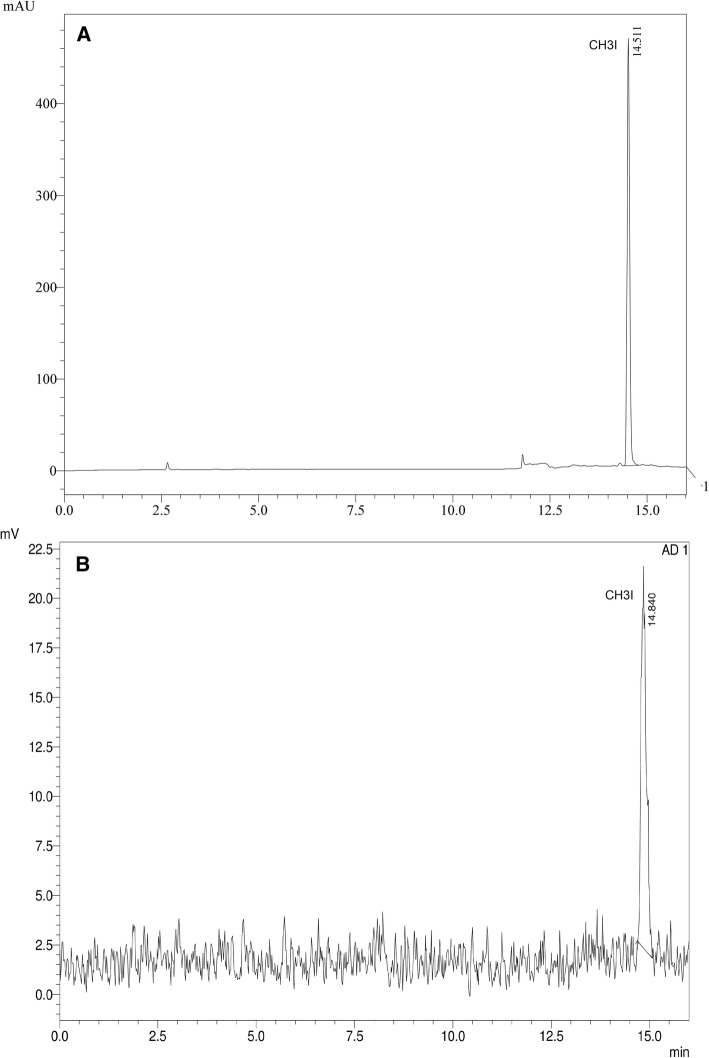
**a**) CH_3_I at 225 nm. **b**) [^11^C]CH_3_I with gamma detector

In these conditions, the peak corresponding to CH_3_I had a retention time of 14.5 min. This was corroborated using the gamma detector by collecting [^11^C]CH_3_I on acetonitrile and injecting in the same conditions (see Fig. 5).

**Fig. 6:**
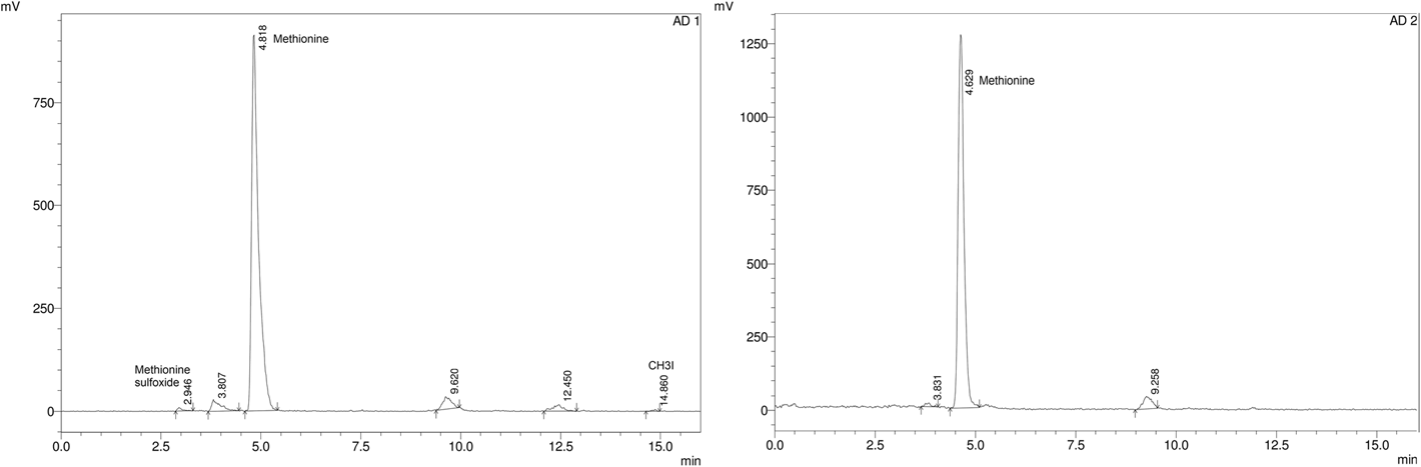
Using the proposed method with gamma detection: **a**) [^11^C]MET. **b**) [^11^C]MET with a second solid phase extraction cartridge

Five radiochemical impurities were observed when analysing the [^11^C]MET sample under these conditions (Fig. 6 a). Two of them had a retention time lower than that of methionine and three of them had higher retention time. Only two could be identified; the first corresponded to the oxidised product of methionine (impurity c), and the last one corresponded to [^11^C]CH_3_I residues.

The proposed method is better because it enabled a greater number of impurities to be determined than those which could be identified using the EP method. Under the conditions proposed by EP or any of the methods described in Table 1, it was not possible to determine the presence of [^11^C]CH_3_I or other lipophilic radiochemical impurities in the final product because the mobile phase is a phosphate buffer. With the EP method, there is a risk of underestimating the radiochemical purity. Pascali et al. (Pascali et al., 1999) used a second extraction cartridge to retain the most lipophilic impurities before the final filter, increasing the radiochemical purity of the final product. We placed, a second solid phase extraction cartridge (Sep-Pak tC18) before the final filter, increasing the radiochemical purity over 90%, considering all the impurities present in the sample (see Fig. 6 b). The purpose of this second cartridge was to trap the more lipophilic impurities formed in the reaction and to the CH_3_I that may have been retained in the first cartridge during the reaction.

The method was validated according to ICH Q2B for UV and gamma detection. (Guidance for Industry, 1997). For UV detection the resolution was: a) 2.0 between L-homocysteine and L-homocysteine thiolactone; b) 3.6 between L-homocysteine thiolactone and L-methionine and c) 6.1 between L-homocysteine and L-methionine. The repeatability (*n* = 10) for the references solutions were: a) RSD = 1,6% (0.204 mg/mL of L-homocysteine), b) RSD = 1,6% (0.213 mg/mL of L-homocysteine thiolactone) and c) RSD = 1,5% (0.201 mg/mL of L-methionine). Linearity was verified in the following intervals: a) 0.204 to 2.10 mg/mL for L-homocysteine, b) 0.064 to 0.66 mg/mL for L-homocysteine thiolactone and c) 0.202 to 2.04 mg/mL for L-methionine. The intermediate precision was checked. The detection limit based on the Standard Deviation of the Response and the Slope were 0.13 mg/mL, 0.04 mg/mL and 0.05 mg/mL for L-homocysteine, L-homocysteine thiolactone and L-methionine respectively. The quantitation limit, also based on the Standard Deviation of the Response and the Slope, were 0.38 mg/mL, 0.11 mg/mL and 0.16 mg/mL for L-homocysteine, L-homocysteine thiolactone and L-methionine respectively.

Linearity of [^11^C]MET was verified for gamma detection in the interval from 19 to 525 MBq/mL.. The intermediate precision was checked. The detection and quantitation limits, based on the Signal-to-Noises of [^11^C]MET, were 0.05% and 0.1% respectively.

### Enantiomeric purity

The method proposed by the EP for the determination of enantiomeric purity is chiral thin-layer chromatography (TLC), which allows the determination of D and L-methionine between the established limits. This is a slow method and presents an inadequate detection due to the decay of the sample.

We proposed a modification of the chiral HPLC method previously described by Rice et al. in order to improve this test (Rice & Yokell, 2017). A chiral Astec CHIROBIOTIC T column of 10 cm × 4.6 mm (5um) was used with a mixture of water and methanol (20:80) at a flow rate of 1 mL / min and gamma and UV detection at 225 nm. Under these conditions, a very good separation was obtained in a run time of 7 min.

**Fig. 7:**
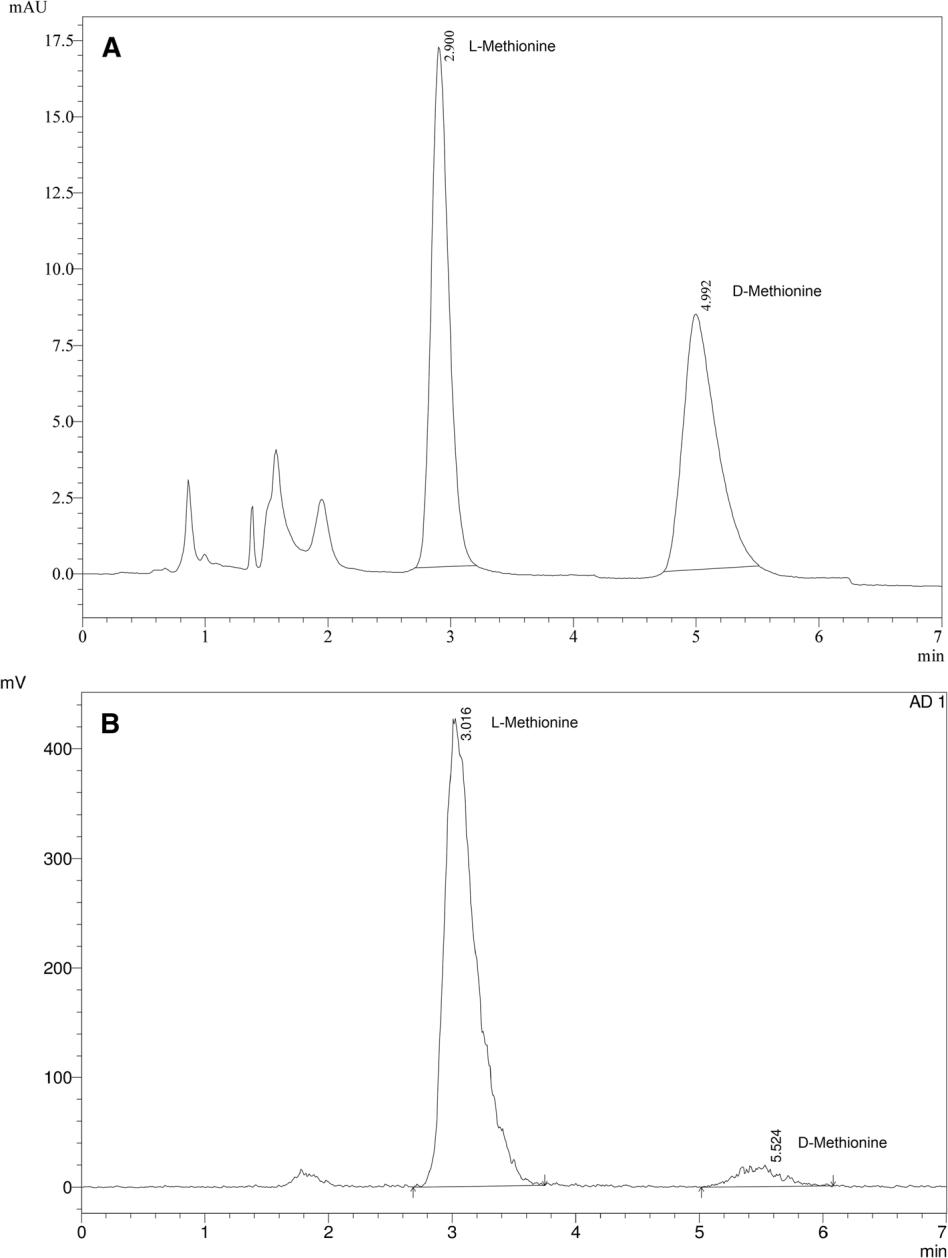
HPLC with the chiral column. **a**) The resolution of the standard D, L Methionine at 225 nm. **b**) The sample using a gamma detector

However, this method requires a second HPLC for the determination of the radiochemical purity. It allows a reliable result to be obtained in a time adjusted to the half-life of the radionuclide. The chromatograms of the standard and the sample are shown in Fig. 7.

## Conclusion

The proposed radiochemical purity method allowed the identification of a greater number of radiochemical impurities compared to the method proposed by the EP monograph, in a period of 16 min.

The proposed enantiomeric purity method enabled to perform the determination in a reasonable amount of time.

A discussion concerning the limit of 95% radiochemical purity proposed by the EP is necessary. If more radiochemical impurities can be detected, it is reasonable to propose a limit of 90% for this parameter to perform clinical examinations.

## Methods

### General methods

The methionine sulfoxide, methionine sulfone, methylene iodide and sodium phosphate monobasic were purchased from Sigma-Aldrich (San Luis, Missouri). For the preparation of the QC HPLC solvent and the phosphate buffer used to elute the reaction mixture of the cartridge, sodium phosphate monobasic was diluted with water to the specified concentration, without adjusting the pH. The sodium hydroxide was purchased from Merck (Darmstadt, Germany). The saline injection (NaCl 0.9%) was supplied by Fármaco Uruguayo Laboratory (Montevideo, Uruguay). Sep-Pak Light C18 and Sep-Pak tC18 Plus Short were obtained from Waters (Milford, MA).

[^11^C]CO_2_ was produced by the 16.5 MeV proton bombardment of a N_2_/O_2_ (1% O_2_ in N_2_) target using an GE PETtrace 800 Series Cyclotron (GE Healthcare, Chicago, Illinois). The [^11^C] CO_2_ was converted to [^11^C] methyl iodide in the gas phase using a GE TRACER FX C Pro module (GE Healthcare, Chicago, Illinois).

Radioactivity was measured using a Capintec (Ramsey, NJ) CRC-25 PET dose calibrator.

### Synthesis of [^11^C]MET

The C18 Sep-Pak Light cartridge was preconditioned with EtOH (5 mL). The EtOH was dried with N_2_ (g) over a period of 15 min. Prior to receiving the activity in the hot cell, the Sep-pak was charged with 2 mg L-homocysteine thiolactone dissolved in 100 uL NaOH:EtOH (1:1). After passing [^11^C]CH_3_I by helium (15 mL/min) through the C18 Sep-Pak cartridge, the reaction mixture was eluted off the cartridge with a phosphate buffer (5.5 mL NaH_2_PO_4_ 0.05 M) and diluted using 4.4 mL NaCl 0.9%. The eluate fraction containing [^11^C]MET was passed through a Millex-GS filter into a final product vial.

Two different C18 cartridges were used: the first one was used for methylation reaction, while the second is for additional purification. The tC18 Sep-Pak cartridge was preconditioned with EtOH (5 mL) and sterile water (10 mL). The remaining water in the cartridge was pushed out using air (10 mL). An additional second cartridge was placed before the final filter in order to increase the radiochemical purity.

### Quality control of [^11^C]MET

The chemical and radiochemical purities, as well as the radiochemical identity of [^11^C]MET and its by-products, were determined using a Shimadzu (Kyoto, Japan) HPLC system equipped with two model LC-20 AD pumps, a model SPD-M20A UV absorbance detector and a flow count (LabLogic, Sheffield, United Kingdom) Na (Tl) I scintillation detector (model B-FC-3200). The operation of the Shimadzu HPLC system was controlled using LC-Solution Shimadzu software. The method was validated according to ICH Q2B for UV and gamma detection.

#### Radiochemical purity

The column was a MACHEREY-NAGEL (Düren, Germany) EC125/4.6 NUCLEODUR 100–5 C18ec (REF 7600002.46). The mobile phase A was KH_2_PO_4_ 1.4 g/L and mobile phase B was acetonitrile; the run was conducted for 16 min, with a gradient 0 to 6 min at 2% acetonitrile and 6 to 16 min at 80% acetonitrile. The flow rate was 1.0 mL/min. Gamma and UV detection at 225 nm were used.

#### Enantiomeric purity

The selected column was an Astec Chirobiotic T column from SUPELCO (Pennsylvania, USA) of 100 × 4.6 mm and 5 μm (Cat. 12022AST). An isocratic mobile phase was used, as was a mixture of water and methanol (20:80). The run time was 7 min, and the flow rate was 1.0 mL/min. Gamma and UV detection at 225 nm were used.
